# Benefit of BK Polyomavirus Screening in the First Year After Lung Transplantation

**DOI:** 10.3389/ti.2025.15265

**Published:** 2025-09-30

**Authors:** Edith Renoult, David Turgeon, Suzon Collette, Francois Coutlée, Charles Poirier

**Affiliations:** ^1^ Division of Nephrology, Department of Medicine, Centre Hospitalier de l’Université de Montréal, Université de Montréal, Montreal, QC, Canada; ^2^ Division of Internal Medicine, Department of Medicine, Hôpital Maisonneuve-Rosemont, Université de Montréal, Montreal, QC, Canada; ^3^ Division of Nephrology, Department of Medicine, Hôpital Maisonneuve-Rosemont, Université de Montréal, Montreal, QC, Canada; ^4^ Department of Microbiology, Infectiology and Immunology, Centre Hospitalier de l'Université de Montréal, Université de Montréal, Montreal, QC, Canada; ^5^ Division of Pneumology, Department of Medicine, Centre Hospitalier de l'Université de Montréal, Université de Montréal, Montreal, QC, Canada

**Keywords:** BK polyomavirus, routine BK polyomavirus DNAemia, lung transplantation, renal failure, early screening

Dear Editors,

Other than case reports of BK polyomavirus-associated nephropathy (BKPyVAN) with end stage kidney disease [1] or BK polyomavirus (BKPyV)-related urothelial carcinoma [2], little is known about the incidence and effects of BKPyV replication in lung transplant recipients (LTR). Most of BKPyV infection data have been obtained from kidney transplant recipients (KTR) [2]. BKPyVAN, a cause of early renal graft failure, is positively correlated with high plasma BKPyV replication [2]. In KTR, early screening for active BKPyV infection together with reduction of immunosuppression have been shown to be effective for preservation of allograft function [2]. However, the effect of BKPyV replication in LTR has been understudied.

We performed a prospective analysis to compare the incidence and kinetics of BKPyV replication between LTR and KTR during the first year after transplantation and to evaluate the course of viral load in LTR.

Of the 310 adult patients who underwent kidney or lung transplantation in Montreal University hospitals (Canada) between January 2018 and January 2020, 195 KTR and 102 LTR had a functioning graft at least 3 months after transplantation and were used as the study population. BKPyV replication monitoring was performed using BKPyV QNAT analysis of plasma samples, as part of routine clinical care, at 1, 2, 3, 6, 9 and 12 months after transplantation.

In LTR, detection of BKPyV replication was not followed by a specific therapeutic intervention but a renal biopsy was scheduled in cases of unexplained kidney dysfunction. In KTR, BKPyV infection was managed by tapering off of the immunosuppressive drugs and regular monitoring for BKPyV-DNAaemia [2].

We examined the incidence, timing and kinetics of BKPyV replication in the LTR cohort and compared the results with those of the KTR cohort in the same period. Occurrence of biopsy proven BKPyVAN, acute rejection, and deaths as well as analysis of the estimated glomerular function rates (eGFR) at 12 and 24 months were evaluated in the LTR group (where BKPyV replication was not managed by reduction of immunosuppression).

The main characteristics and the outcomes of the patients are detailed in Table 1. The incidences of BKPyV replication during the first year after transplantation in LTR and KTR was 17% and 30.25% respectively. BKPyV replication occurred within the first 3 months after transplantation in 94% of LTR + and 64% of KTR +. The median peak viral load was 422 copies/mL (range 23–79683 copies/mL) in LTR with BKPyV replication (LTR +) and 4770 copies/mL (range 26–954,000 copies/mL) in KTR with BKPyV replication (KTR +) (p = 0.0258). Viral load reached 10,000 copies/mL or more, in 23% of LTR+ and 38.9% of KTR+ during the first 12 months.

**TABLE 1:** Characteristics of patients who received a lung transplant or a kidney transplant between January 2018 and January 2020^a^, incidences of BKPyV-DNAemia in the first year after transplantation and outcomes after transplantation.

Variables	Transplant received							
	Lungs				Kidney			
	All (n = 102)	BKV - (n = 85)	BKV + (n = 17)	P value	All (n = 195)	BKV – (n = 136)	BKV + (n = 59)	P value^b^
Recipient characteristics								
Age (y) median (IQR)	58 (18–73)	56 (18–73)	61 (18–73)	0. 30	55 (22–76)	54.5 (22–76)	55 (24–73)	0.14
Sex, male (%)	70.5	71.76	64.7	0.56	66	66.9	66.1	
Donor characteristics								
Age (y) median (IQR)	45 (14–84)	45 (14–84)	51 (24–68)	0.43	52 (10–77)	52 (10–77)	51 (24–68)	0.56
Sex, male (%)	56.8	60	41.1	0.18	64.6	59.5	76	0.03
Living (%)	0	0	0		19.4	20.5	16.9	0.69
Induction IS								
with ATG (n)	0	0	0		50	36	14	0.72
with Basilixumab (n)	46	31	15	0.0001	145	100	36	0.72
Maintenance IS								
with TAC/myc.a/cort (n)	95	79	16	1	194	135	58	0.51
CMV Rneg/Dpos^c^ Ureteral stent placed	25 0	21	4	1	39 195	30	9	0.39
Acute rejection in the 1st year	6	6	0		21	9	12	0.009
eGFR (ml/min/1.73 m^2^)								
After 1 year	68.44 ± 26.35 (n = 94)	68.56 ± 26.40 (n = 77)	67.88 ± 26.92 (n = 17)	0.96	55.57 ± 21	56.18 ± 21	54.19 ± 20	0.55
After 2 years	63.92 ± 25.94 (n = 90)	67.51 ± 25.06 (n = 74)	56.56 ± 29.39 (n = 16)	0.21				
Proven BKPyVAN	0	0^d^	0					

Abbreviations: CMV,cytomegalovirus; ATG, antithymocyte globulin; R, recipient; D, donor; TAC, tacrolimus; myc.a, mycophenolic acid; cort, corticoids; neg, negative; pos, positive; eGFR, estimated glomerular filtration rate; BKV +, with plasma BKPyV replication; BKV -, without plasma BKPyV replication.

^a^ This study was approved by the University of Montreal Institutional Review Board.

^b^ Descriptive statistics were calculated with categorical data reported as counts and percentages, continuous data as means ± standard deviations if normally distributed and medians with ranges if non-normally distributed. P values between BKPyV negative (-BKV- -) and BKPyV positive (-BKV+-) groups of <0.05 were considered statistically significant.

^c^ All cytomegalovirus (CMV) seronegative recipients transplanted with organs from CMV seropositive donors received prophylaxis for 3–6 months after transplantation with intravenous ganciclovir or valganciclovir.

^d^ One lung recipient of the BKV- group was diagnosed with biopsy-proven BKPyVAN in the fifth year after transplantation, with unexplained sub acute renal failure (eGFR, of 42 mL/min) and a plasma BKPyV load of 302,000 copies/mL.

One year after transplantation, BKPyV-DNAemia persisted in nine of the LTR+ (median 2290 copies/mL, range 23–126 240 copies/mL). No LTR developed unexplained kidney dysfunction. The eGFR did not differ between LTR+ and LTR- (without BKPyV replication) groups at 12 months and 24 months after transplantation. Four deaths occurred, in the LTR -. LTR+ did not differ from LTR- with respect to age, immunosuppression, and cytomegalovirus serostatus.

BKPyV replication is an important cause of kidney dysfunction in the first year after kidney transplantation [2]. Studies reporting the results of screening for BKPyV after lung transplantation are rare and often cross sectional [3–5]. Doucette et al [6] monitored BKPyV replication in urine and plasma samples from 28 LTR at 3, 6, and 9 months post-transplantation and reported no detectable BKPyV-DNAemia in any subject and BKPyV-DNAuria in 17.8% of LTR. In our larger prospective study performed within 102 LTR, at 1, 2, 3, 6, 9, and 12 months after transplantation, monitoring demonstrated BKPyV replication in 17%. This value is high, but less that of our kidney transplant group (30. 25%) studied with the same molecular assay, during the same period when LTR and KTR were under tacrolimus, mycophenolic acid and corticosteroids. BKPyV replication was detected early after transplantation, in both groups: the first positive case of BKPyV-DNAemia was observed in the first trimester in LTR and in KTR. The rate of replication was lower in LTR than in KTR.

While the diagnosis of BKPyV replication was not followed by changes in the immunosuppression regimen in LTR+, the viral load reached more than 10,000 copies/mL, a value that correlates strongly with presumptive BKPyVN in KTR [2]. A kidney biopsy was not systematically performed on LTR + patients. The renal functions of the LTR+ and LTR- patients were not different at 1 or even 2 years after transplantation. Interestingly, one LTR- subject developed renal dysfunction with biopsy-proven BKPyVAN 4 years after transplantation.

The conditions that lead to the development of BKPyVAN from BKPyV replication may differ between KTR and LTR. Some factors are specific to the kidney transplant: the graft (major reservoir of latent BKPyV) may transmit the virus [2]. Acute tubular damage due to early intra-renal immunological and ischemic reperfusion lesions [7], and the placement of ureteral stent during kidney transplant surgery [8] may play a role in the development of BKPyVAN. BKPyV-DNAemia, a surrogate marker of BKPyVAN in kidney transplantation [2] has not be established as such in lung transplantation. Whether there is a lung transplant specific timeline of BKPyVAN remains unclear [9] Recently, Dube et al [1] reported 11 cases of biopsy proven BKPyVAN in LTR presenting with unexplained kidney dysfunction, at a median of 46 months after transplantation. In KTR, before screening protocols were available, most cases of BKPyVAN were diagnosed, in the first year after transplantation, in patients with kidney failure [10].

Based on the results of our prospective, observational study conducted on 102 LTR, routine BKPyV-DNAemia surveillance during the first year after lung transplantation is unlikely to be beneficial as it is in kidney transplantation [2]. To detect BKPyVAN early and preserve renal function, future investigations should focus on the benefit of routine screening (yearly?) after the first year of lung transplantation. Nonetheless BKPyV-DNAemia should be tested upon suspicion of kidney dysfunction (at any time after transplantation) for the diagnosis of BKPyVAN.

## Data Availability

The original contributions presented in the study are included in the article/supplementary material, further inquiries can be directed to the corresponding author.
